# Rare Presentation of Gerbode Defect in a 4-Month-Old Nigerian and a Review of the Literature

**DOI:** 10.1155/2013/564786

**Published:** 2013-12-25

**Authors:** Barbara E. Otaigbe, Douye Orubide

**Affiliations:** Paediatric Care Hospital, 8c Elelenwon Street, GRA Phase 2, Port Harcourt, Rivers State, Nigeria

## Abstract

A Gerbode defect is a very rare congenital anomaly which presents as a direct or an indirect type. We report a 4 month old Nigerian child who presented with poor feeding and failure to thrive and whose echocardiography confirmed an indirect Gerbode with a direct left ventricle to right atrial shunt due to adherent leaflets of the tricuspid valve. This was confirmed by surgery done outside the country.

## 1. Introduction

The Gerbode ventriculoatrial defect is a very rare congenital anomaly [1]. It is described as a communication between the left ventricle and the right atrium. There are 2 types known, a direct and an indirect as reported by Gerbode et al. [2]. In a patient with a perimembranous ventricular septal defect (VSD) and a defect in the tricuspid valve, the shunt is from left ventricle to right ventricle then through the tricuspid valve into the right atrium. The communication thus occurs below the tricuspid valve. This is referred to as an indirect left ventricular—to—right atrial shunt while in a true or direct Gerbode, the blood in the left ventricle goes through the small area of the membranous septum where there is the potential for a left ventricular—to—right atrial shunt. This communication is above the tricuspid valve.

There are contrasting reports of this defect being first reported in 1857 by Meyer [3] and by Thurman in the 19th century [4]. The first successful closure of such a defect was reported by Kirby et al. (using hypothermia and inflow occlusion) at the Hospital of the University of Pennsylvania in 1956 [5]. Gerbode (after whom the defect was named) was a noted surgeon at Stanford University who successfully operated on a series of patients with a left ventricular—to—right atrial shunt in 1958 [2]. According to the STS Congenital Heart Nomenclature and Database Project [6], the definition of a Gerbode defect is a true left ventricular—to—right atrial communication.

We report a rare form of Gerbode defect in which a perimembranous VSD is restricted by the adherent septal and anterior tricuspid leaflet at the anterocommisural area resulting in an LV to RA jet, though through the tricuspid valve.

## 2. Case Report 

A four-month-old female infant was brought to a private paediatric hospital in Port Harcourt, Nigeria; by her mother with complaints of poor feeding and poor weight gain, weighing 4.5 kg (<3rd percentile) at presentation and 2.8 kg at birth.

There was no history of fast breathing, cough, cyanosis; or loose excessive sweating. She is the only child of a nonconsanguineous couple delivered by emergency caesarean section at term for cord around the neck. Mother denies intake of any herbs or drugs in early pregnancy. Father died after a road traffic accident during index pregnancy.

On examination, she had a left precordial bulge. Her heart rate was 140 b/min. Apex beat was at the 4th left intercostal space, midclavicular line. She had a grade 3/6 ejection systolic murmur. The patient was in mild respiratory distress with a respiratory rate of 64 cycles/min. There was no hepatomegaly.

Chest X-ray showed situs solitus, levocardia, mild cardiomegaly; and pulmonary plethora. ECG showed normal sinus rhythm, extreme right axis deviation; and left ventricular hypertrophy.

**Figure 1:**
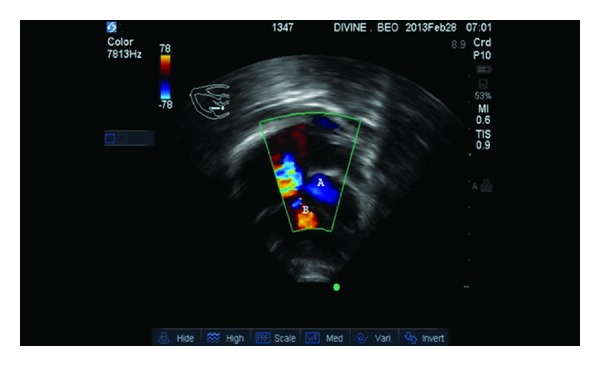
Color flow Doppler from an apical four-chamber view showing systolic flow from LV to RA though the adherent septal and anterior tricuspid leaflets. Note the Gerbode jet (A) and the VSD jet (B).

**Figure 2:**
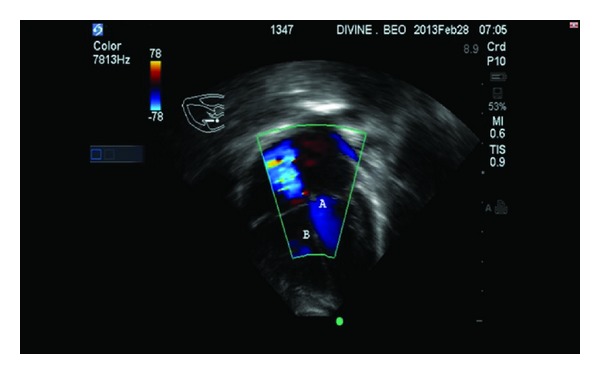
Color flow Doppler showing the tricuspid leaflet with no tricuspid regurgitation. (A) indicates LV. (B) shows the RV.

Echocardiography revealed a dilated left atrium and a hypertrophied left ventricle. There was a left ventricular—right atrial shunt through an aneurysmal septal leaflet of the tricuspid valve restricting the perimembranous VSD. Effective perimembranous VSD size was 7 mm. The LV to RV to RA mean velocity was 50.5 cm/s and TV gradient was 50 mmHg.  Figure 1 is color flow Doppler of a 4-chamber view showing systolic flow (A) into right atrium from the adherent septal and anterior leaflets of the tricuspid valves and (B) shows restricted 7 mm perimembranous VSD shunting left to right.  Figure 2 is color flow Doppler showing the tricuspid leaflet with no tricuspid regurgitation. A diagnosis of acyanotic congenital heart disease; Indirect Gerbode Defect; was made.

The mother was informed and counseled extensively on the need for urgent surgical repair of the defect. Mother was indigent and because of the need to confirm this rare defect, funds were solicited for on behalf of the child and she was sent to India for surgery.

A repeat echocardiogram in India reported an indirect Gerbode phenomenon through the tricuspid valve with LV to RV to RA jet and a perimembranous juxtatricuspid VSD with membranous septal aneurysm formed by the anteroseptal tricuspid commissural area. The effective VSD ventricle from the left atrium measures around 7 mm with TV gradient of 50 mmHg.

Findings at surgery were a perimembranous VSD measuring about 7-8 mm in diameter restricted by the adherent septal and anterior leaflets at the anteroseptal commissural area resulting in a direct RV to RA jet. Patient had a ministernotomy with transatrial Gortex patch closure of perimembranous VSD and closure of the defect between the anterior and septal leaflets of the tricuspid valve.

## 3. Discussion

Gerbode defects are caused by anatomic deficiency of the membranous septum which is divided into two anatomic portions depending on to the relationship to the septal leaflet of the tricuspid valve. There is the more apically located *interventricular portion* and the more basally placed *atrioventricular portion*. The tricuspid valve usually attaches to the membranous septum about 1 cm apical to the attachment of the mitral valve; thus the atrioventricular septum separates the left ventricle from the right atrium [7]. Another way of describing the defect is by using the classification into the supravalvular and infravalvular defects by Riemenschneider and Moss [8]. In this classification based on the anatomical relationship of the LV to RA shunt with the tricuspid valve, the supravalvular defects are in the atrio-ventricular septum while the infravalvular defects occur between the left and right ventricles and then to the right atrium through a defect in the tricuspid valve. These valve defects can be due to leaflet perforations, malformation, widened commissure; or clefts.

The Gerbode defect is a ventriculo-atrial defect, causing oxygenated blood from the left ventricle to jet into the right atrium at a high velocity as seen in our patient with a mean velocity of 50.5 cm/sec and gradient of 50 mmHg. The large systolic pressure gradient between the left ventricle and the right atrium is most likely the cause of the high velocity systolic Doppler flow signal. A high Doppler gradient is one of the hallmarks of the Gerbode ventriculo-atrial defect because of the difference between the left ventricular systolic pressure and the low right atrial pressure [7].

The direct Gerbode defect is rarer than the indirect and occurs in the membranous part of the ventricular septum above the tricuspid valve and allows blood to shunt from the left ventricle directly into the right atrium [8].

The indirect Gerbode defect which is seen in this patient, though the commoner form of the defect in which there is a ventricular septal defect with blood moving from the left to right ventricle then through a defective tricuspid valve into the right atrium; is also rare and has not been previously reported in an African child. Rarer still is the fact that the defect is through adherent leaflets of the tricuspid valve.

In both defects, left ventricular outflow tract to right atrium communication allows for shunting of blood to the right atrium during systole. If this communication is large, it can lead to volume overload and chamber enlargement as seen in our patient [9].

This patient presented with failure to thrive and was not in congestive heart failure; however in a series [7] of six patients (2 males and 4 females) who underwent closure of a direct congenital Gerbode-type ventriculo-atrial defect between the years 1990 and 2008 at Children's Memorial Hospital, three had congestive heart failure, 2 had failure to thrive, and 2 had exercise intolerance. The age range was 0.4 to 19 years. The size of the ventriculo-atrial defect ranged from 4 to 8 mm, with a mean size of 6.2 ± 2 mm; while in our patient the Gerbode defect was 6 mm and the perimembranous VSD was 7 mm.

There is a similar report in which a small perimembranous VSD was completely covered by an elongated sail-like anterior tricuspid leaflet forming an aneurysm and directing the shunt into right atrium [10].

Spontaneous closure of these defects is very rare and surgical repair is excellent [7].

Though this patient presented relatively early with no overt heart failure symptoms or signs she already had mild pulmonary hypertension. The risk of heart failure and eventual pulmonary hypertension is high and this child with a mother who is an indigent widow was a candidate for pulmonary hypertension but for timely intervention.
